# The effects of Kinesio tapes on facial swelling following bimaxillary orthognathic surgery in the supraclavicular region

**DOI:** 10.1186/s40902-023-00385-7

**Published:** 2023-06-19

**Authors:** Mohsen Golkar, Anita Taheri, Mostafa Alam, Yasin Asadi, Seied Omid Keyhan

**Affiliations:** 1 School of Dentistry, AJA University of Medical Sciences, Tehran, Iran; 2 Department of Oral and Maxillofacial Surgery, School of Dentistry, Shahid Beheshti University of Medical Sciences, Tehran, Iran; 3 School of Dentistry, Shahid Beheshti University of Medical Sciences, Tehran, Iran; 4 Department of Periodontics, School of Dentistry, AJA University of Medical Sciences, Tehran, Iran; 5 Department of Oral & Maxillofacial Surgery, Gangneung-Wonju National University, Gangneung, South Korea; 6 Department of Oral & Maxillofacial Surgery, College of Medicine, University of Florida, Jacksonville, FL USA; 7 Maxillofacial Surgery & Implantology & Biomaterial Research Foundation (www.Maxillogram.com), Tehran, Iran; 8 Iface Academy, Marietta, GA USA

**Keywords:** Kinesio tapes, Facial swelling, Orthognathic surgery, Supraclavicular region

## Abstract

**Background:**

Several osteotomies are required for orthognathic surgery to reposition the jaws correctly. This study aimed to evaluate whether Kinesiotaping can reduce swelling, pain, and trismus following orthognathic surgery of the facial skull.

**Materials and methods:**

The present study consists of two phases. In the split-mouth phase, 16 skeletal class III patients underwent Bimax Orthognathic surgery, and Kinesiological tape (KT) was applied on one half of the face. In the prospective case–control phase, 30 patients were divided into two groups. Kinesio tape was applied on both sides of the face of the Kinesio group, and pressure dressing and ice therapy were used for the second group. The tape was parallel to the lower border of the mandible along its entire length, tangent to the labial commissure area on the studied side. The tape was placed in place for 5 days. Edema was evaluated by measuring the distance from the menton to the lower edge of the tragus. The maximum mouth-opening trismus was evaluated, and the VAS index was used to evaluate pain.

**Results:**

There was evidence of swelling reduction after KT; within the same study, differences between the left and right sides as well as for the same side were statistically significant (*p* < 0.001). As a result of tapping lymphatic Kinesio tape on the affected area, tension was reduced, and lymphatic circulation was restored. Blood and lymph microcirculation was improved, enabling the body to heal itself.

**Conclusion:**

Kinesio tape reduced swelling after orthognathic surgery in a positive way. As a simple, non-traumatic, economical method, Kinesio taping seems promising.

## Introduction

An orthognathic surgeon must perform several osteotomies to reposition the jaws correctly. Mucoperiosteal flaps resulting in full-thickness osteotomies may cause swelling postoperatively. In addition to complicating surgery and the patient’s recovery, swelling can also restrict the patient's airway [1–5]. Several methods have been described and used to manage the immediate inflammatory response after head and neck surgery, including analgesics, corticosteroids, antibiotics, protease enzymes, lasers, cryotherapy, or manual lymphatic drainage [1, 6–13]. Swelling, pain, and trismus cannot be prevented or reduced without undesirable side effects [14]. Hence, alternative techniques for treating orthognathic surgery patients should be developed to reduce pain, swelling, and trismus. Kinesiological tape (KT) has become increasingly common in treating sports injuries and other ailments since its introduction in the 1970s. In addition to strengthening muscles and joints, KT is claimed to relieve pain and increase blood flow. Despite these claims, limited evidence supports them, and further research is needed [15, 16]. Lymphedema treatment with KT has become more prevalent in recent years. With its weight similar to the epidermis, the tape lifts the skin, improves blood flow, and relieves lymph drainage and hematoma congestion. Through space provision, fluid is directed from areas with more pressure to areas with less pressure, and these strips are used to drain lymph [17]. Inflammatory reactions or hematomas in the head and neck can be drained more quickly using this technique following surgery. Post-operative trismus and swelling following orthognathic surgery can frustrate patients, as they are often socially and professionally isolated [18].

Despite its increasing use in rehabilitation and injury prevention protocols, there is no clear evidence of the mechanisms behind the beneficial effects of KT. Several promising studies and reports have been presented, but more evidence-based studies are needed to prove clinically beneficial effects [19]. On the whole, however, KT has been shown to have the potential to reduce swelling and hematoma [15]. KT tapes consist of elastic linen tape with an adhesive layer that is non-allergenic. It can provide 30–40% longitudinal traction. Stretches and rotations of the muscles stretch the skin of the treated area. A slight stretch is applied to the skin with KT. The tape length reverts to its original position when the limbs return to their starting points. This produces convolution under the adhesive area by creating a pulling force on the skin. Blood flow and lymph production are increased due to these complications because they increase the interstitial space between the skin and the connective tissues beneath it. In an animal study, the emerging wrinkles caused the skin to rise and not dent inward. Researchers could promote lymph flow by opening the microvalves in the primary lymph vessels [20]. To obtain reliable measurements, it is not easy to assess facial volume reduction. It has been attempted to measure the accuracy of many methods, but many are inaccurate, complicated, costly, or difficult to standardize [21–32]. The measurement of facial soft tissue has traditionally been performed by probing the needle and measuring its penetration depth [21, 33]. Several imaging-based methods have been reported [22, 23]. Additionally, imaging and analysis with three-dimensional methods have been suggested. However, the correlation between anthropometric skin measurements and three-dimensional volumetric measurements is high, and these measurements are reliable and valid in studies. The present study aimed to investigate the effect of KT on swelling and its reduction, as well as trismus and pain after orthognathic orthodontic surgery, by using two methods: split mouth and case–control, with direct measurements on the skin (anthropometric), to evaluate edema; it is also intended to estimate the MMO and VAS pain index, which will indicate its use in the traumatic jaw and facial injuries. Maxillofacial patients can benefit from KT by reducing morbidity and mortality.

## Methods and material

### Patients

In the present study, a randomized controlled clinical trial evaluating the effectiveness of KT in reducing inflammatory processes for patients undergoing orthognathic surgery was conducted in two phases, a split-mouth phase and a prospective control case phase. The study was approved by the Medical Ethics Committee of Shahid Beheshti University of Medical Sciences (IR. SBMU.RIDS.REC.1400.142). The primary phase of the study included 16 patients with facial deformities who underwent orthognathic surgery at Taleghani and Imam Hossein hospitals in Tehran. Each participant signed an informed consent form before any surgery or treatment.

### Inclusion criteria

All patients should be in good health and avoid using medications daily. They had no pre-existing medical conditions and were not taking medications that could adversely affect their ability to undergo surgery or heal from post-operative wounds. All patients underwent the same protocol and type of osteotomies.

### Exclusion criteria

Patients with regular drug therapy, mental illness, blood clotting disease, diabetes, or chronic infection were excluded from the study. Drugs, alcohol, or nicotine addiction were not permitted. Participants with more than 2 mm asymmetry at the maxilla, mandible, or chin were eliminated from the study.

### General treatment instructions

Preoperatively, intraoral disinfection was conducted using a mouthwash with 0.12% chlorhexidine, while extraoral disinfection was performed by topical application of 1% betadine. The maxillofacial surgery service performed the same standard protocol on all patients. Under sterile conditions, the surgery followed standard procedures (Lefort I in maxilla to advance and BSSO in mandible to setback). Dissolving sutures were used to seal the wound (3–0, Vicryl). (3–0, Vicryl). To relieve pain, 1 g of paracetamol IV (PRN) was given every 8 h until discharge and converted to oral paracetamol. Eight milligram dexamethasone IV half an hour before surgery and every 8 h on the first day, every 12 h on the second day and a dose on the third day, antibiotics 1 g Keflin IV half an hour before surgery and then every 6 h until discharge should be added. Then, 500 mg oral Cephalexin for up to a week, 0.12% chlorhexidine and normal saline for gentle mouthwash every 8 h for 7 days, and sinus congestion control medications containing 25% phenylephrine drops (up to 3 days every 8 h). Antihistamine tablets (up to 1 week after discharge) and two drops in each nostril of normal saline 65% (up to 7 days after discharge, two drops in each nostril every 8 h) were prescribed.

**Fig. 1:**
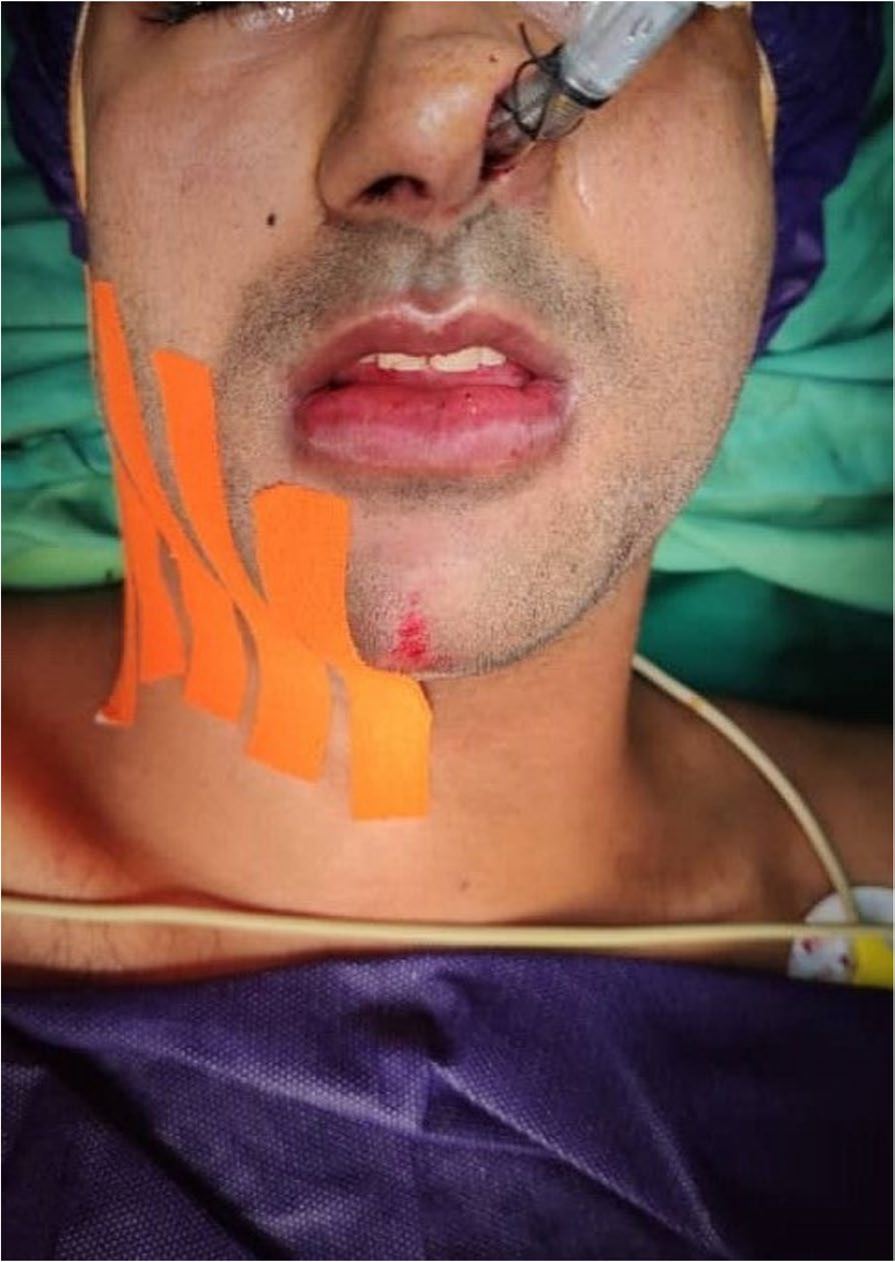
Kinesio tape application

Patients were encouraged to use a cold compress for the first 24 h following surgery, wear a two-way compression bandage for 48 h, and eat a soft, high-protein, high-calorie drink for up to 4 weeks. They should take 24 h of bed rest, brush softly, and maintain proper dental hygiene. In the first phase of the study, KT was used in the immediate post-operative period on the side of the face standardized as the case group, while no KT was used on the side defined as the control (No KT). We used Microsoft Excel and simple random allocation algorithms to decide where the case and control sides should go. In the second phase, two groups of 15 patients were enrolled in the study second phase, matched in terms of age, sex, and anesthetic duration. After surgery, the case group received bilateral KT, whereas the control group received regular medication protocol and cooling therapy. The tapes utilized in this study were made of linen and polyurethane. They were made without the use of any medicines. The band was tangential to the lower mandibular border over its entire length, from the lip commissure area on the examined side to below the earlobe. It was divided into five strips, each 5 cm long and 1 cm thick. The strips were applied to clean, dry skin. The tape was applied from the lower border of the mandible to the bottom (Fig. 1) and stayed for 5 days.

### Measurements

The edema was measured using bilateral measuring strips. To calculate the measurements, we measured the Distance between the menton and the lowest part of the ear lobe. Following the Markovich and Todorovich methods, we calculated the edema ratio (Ec) from preoperative and post-operative measurements using the following formula:

$$\mathrm{Ec}=\mathrm{Post}-\mathrm{Op}\;\mathrm{Distance}\;-\;\mathrm{Pre}-\mathrm{Op}\;\mathrm{Distance}/\mathrm{Pre}-\mathrm{Op}\;\mathrm{Distance}\;\times\;100$$

A calibrator analyzed recorded values for edema at six specific time intervals: before surgery (P), immediately after surgery as baseline (T0), as well as 24 (T1) and 48 (T2), and 120 (T3) h, and 14 days (T4) after surgery. Trismus was determined by measuring the maximum mouth opening (MMO) between the incisal edge of the upper and lower central teeth. The electronic analog index (mobile interactive clinic software) was utilized to assess the severity of pain. In the study’s first phase, edema was assessed, and trismus, pain, and edema were compared in the second phase.

## Results

**Fig. 2:**
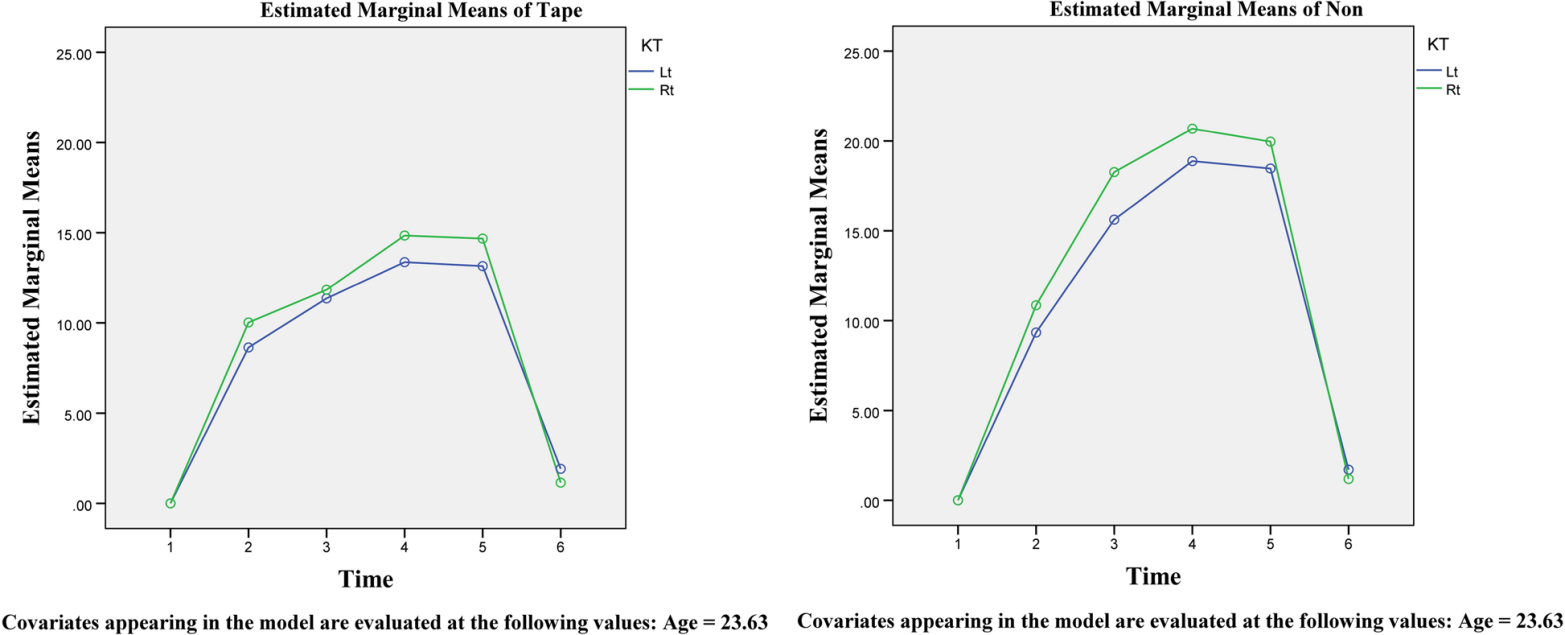
Diagram of changes in edema levels in patients with and without using Kinesio tape

The initial trial involved sixteen patients, including eight men and eight women. Kinesio tape was placed on one of the supraclavicular locations on the right or left (4 + 4 in each sex group). The average operation time for these participants was 231.4 min, with no significant differences between groups. The amount of edema on both sides of the patient's face was measured, and it was shown that the amount of edema based on the calculation of the Markovich and Todorovich method mentioned earlier, at intervals of 1, 2, and 5 days after surgery was significantly less in the side of Kinesio tape than the opposite side. These results were independent of patients' faces (right or left). In addition, in the periods before and immediately after surgery and after 2 weeks of surgery, among the same groups, there was no significant difference in edema between the two sides. In each group using or not using Kinesio tape, the average amount of edema on the right side of the face immediately after surgery, 1, 2, and 5 days after surgery, is higher than on the left. However, statistically, this difference was not significant. The above results can be seen in Table 1 and Fig. 2.

**Table 1:** Edema measured by the Markovich and Todorovich methods on two sides of the face at different times compared

Paired Samples Test									
Paired Differences									
					95 % Confidence interval of the difference				
		Mean	Std. Deviation	Std. Error Mean	Lower	Upper	t	df	Sig. (2-tailed)
Pair 2	ECt0kt-ECt0	-0.76303	1.91066	0.47767	-1.78115	0.25509	-1.597	15	0.131
Pair 3	ECt1kt-ECt1	-5.35272	2.93392	0.73348	-6.91610	-3.78935	-7.298	15	0.000
Pair 4	ECt2kt-ECt2	-5.67926	3.68687	0.92172	-7.64386	-3.71467	-6.162	15	0.000
Pair 5	ECt3kt-ECt3	-5.30501	3.90077	0.97519	-7.38358	-3.22644	-5.440	15	0.000
Pair 6	ECt4kt-ECt4	0.08339	0.93822	0.23455	-0.41655	0.58334	0.356	15	0.727

**Fig. 3:**
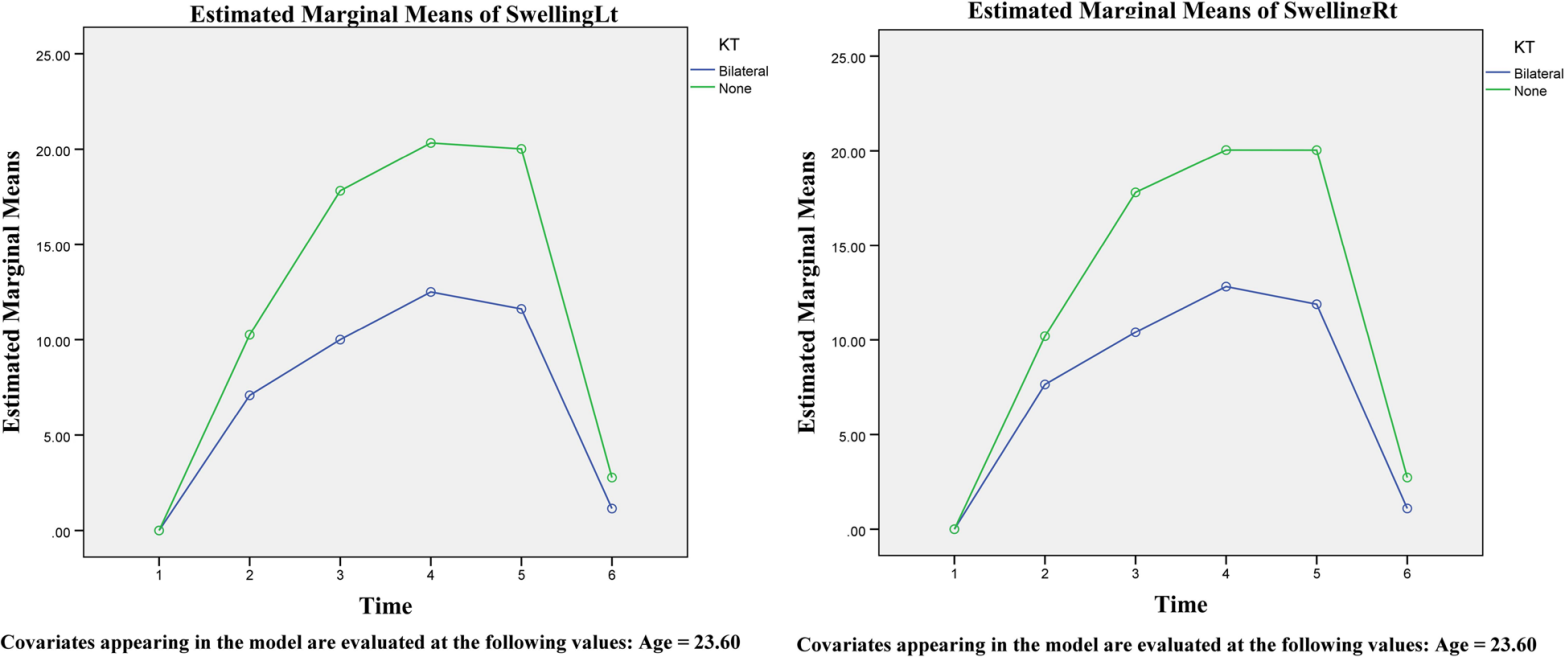
An assessment of the edema levels on the right and left sides of the patient’s face using Kinesio tape at different times

**Fig. 4:**
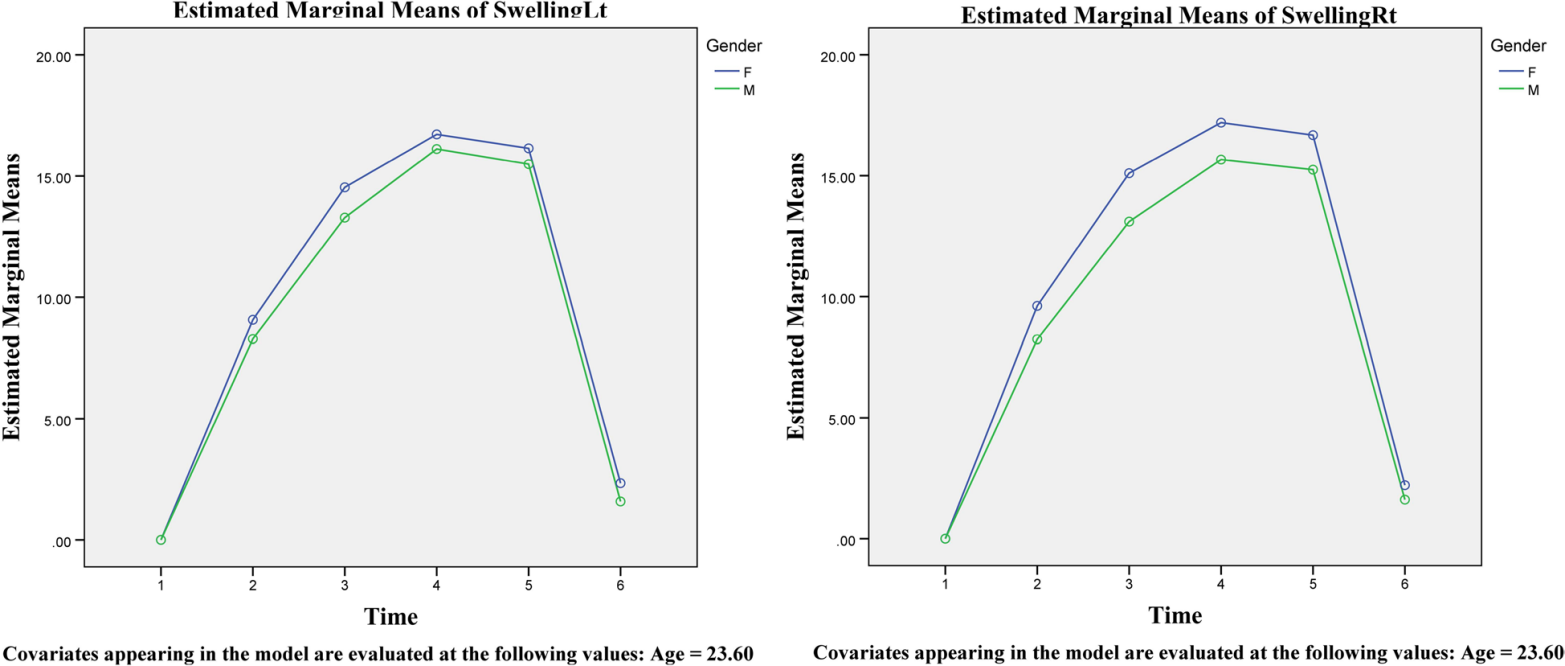
A comparison of the amount of edema on the right and left sides of the face at different intervals in women and men

**Fig. 5:**
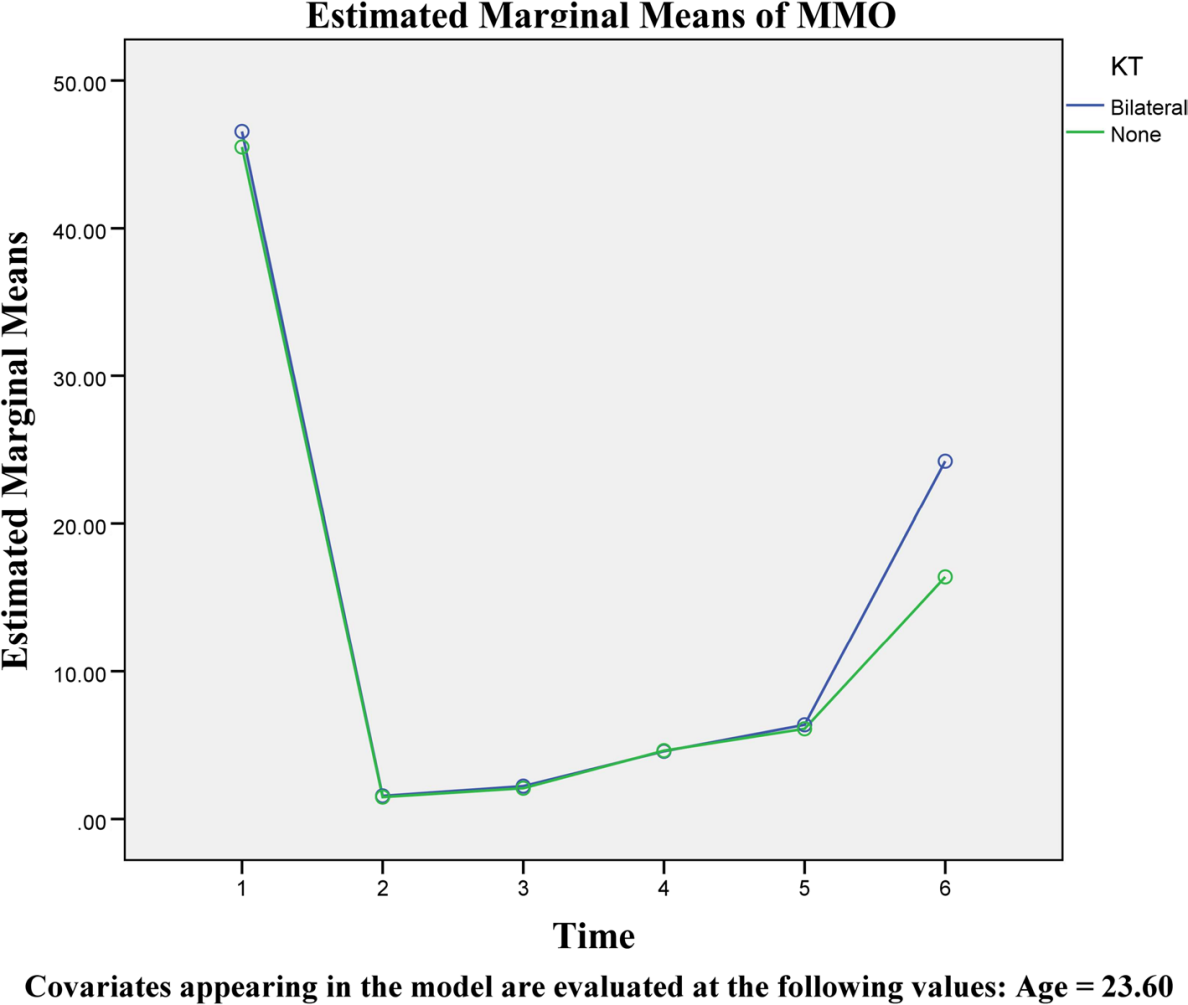
Average MMO with and without Kinesio tape at different intervals

**Fig. 6:**
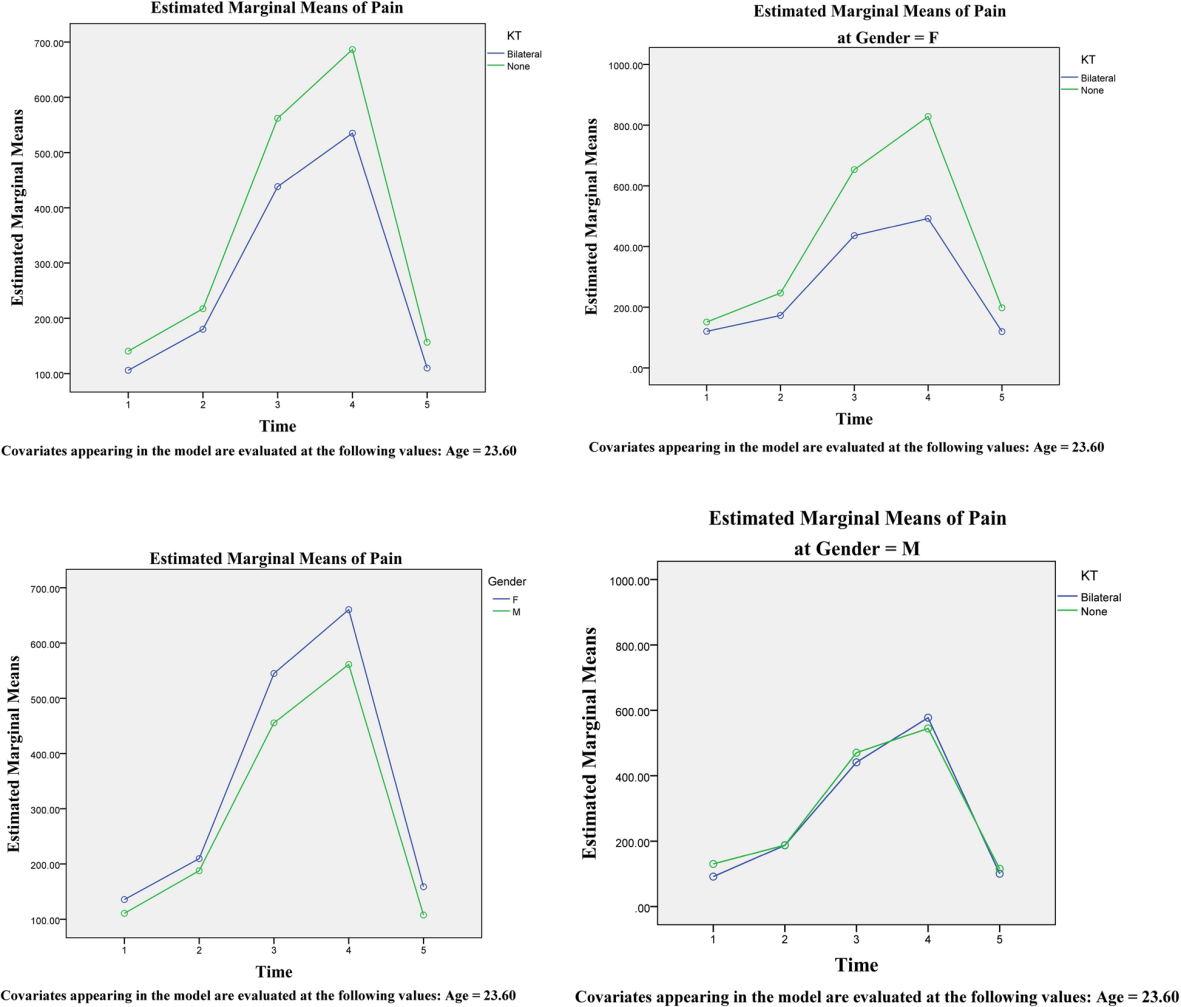
Analysis of pain levels reported over different periods and by different genders

In the second part of the study, 30 patients were included. These individuals included 14 men (7 + 7) and 16 (8 + 8) women, each divided into bilateral use or non-use of Kinesio tape groups. The average surgery time for these participants was 245.1 min, with no significant differences between groups. According to the findings, individuals who utilized Kinesio tape bilaterally had a much-decreased rate of edema (Table 2 and Figs. 3 and 4). MMO also increased more quickly in these patients after 5 to 14 days. These results were obtained regardless of age or gender. On the other hand, facial edema of female patients on the right side is more significant than in male patients, and this issue was statistically significant (Fig. 5). Furthermore, using the visual analog index (VAS) to measure the pain described by patients revealed a reduction in discomfort at various stages following surgery; however, this reduction was not significant except in distinct sex groups (Table 3 and Fig. 6).

**Table 2:** Comparative analysis of the amount of edema in different groups with repeated measurements over time

Source		Type III Sum of Squares	df	Mean Square	F	Sig.
Time	SwellingRt	126.078	5	25.216	22.963	<0.001
	SwellingLt	110.873	5	22.175	18.483	<0.001
	MMO	920.901	5	184.180	18.322	<0.001
Time/Age	SwellingRt	4.469	5	0.894	0.814	0.542
	SwellingLt	2.905	5	0.581	0.484	0.788
	MMO	92.385	5	18.477	1.838	0.110
Time/KT	SwellingRt	457.152	5	91.430	83.263	<0.001
	SwellingLt	498.773	5	99.755	83.146	<0.001
	MMO	356.113	5	71.223	7.085	<0.001
Time/Gender	SwellingRt	19.059	5	3.812	3.471	0.006
	SwellingLt	5.879	5	1.176	0.980	0.433
	MMO	64.889	5	12.978	1.291	0.272
Time/KT/Gender	SwellingRt	6.897	5	1.379	1.256	0.287
	SwellingLt	13.586	5	2.717	2.265	0.052
	MMO	47.692	5	9.538	0.949	0.452

**Table 3:** Comparative analysis of reported pain levels across groups and over time

Source		Type III Sum of Squares	df	Mean Square	F	Sig.
Time	Sphericity	134713.5021	4	33678.376	2.426	0.053
	Assumed					
Time/Age	Sphericity	9471.204	4	2367.801	0.171	0.953
	Assumed					
Time/KT	Sphericity	89460.173	4	22365.043	1.611	0.177
	Assumed					
Time/Gender	Sphericity	37202.467	4	9300.617	0.670	0.614
	Assumed					
Time/KT/Gender	Sphericity	162057.654	4	40514.413	2.919	0.025
	Assumed

## Discussion

The present study showed that using Kinesio tape in the supraclavicular region can reduce morbidity after surgery, particularly edema and trismus of the masticatory muscles. These results are in line with the conclusion of Jaron et al. The effect of Kinesio tape on morbidity after wisdom tooth surgery was investigated in comprehensive review studies published in 2020 and 2021 [34, 35]. Numerous studies have been conducted on edema control postoperatively in maxillofacial surgery. Laser therapy has emerged as a relatively new strategy for decreasing post-operative discomfort, especially edema, in third molar surgery [36–38]. By reducing the permeability of blood vessels, laser radiation is believed to increase lymphatic vessel density and diameter. Maxillofacial surgery, however, has yet to be extensively studied for its application.

There are many benefits to using ice therapy, including its simplicity, affordability, and reproducibility. As a result of changes in blood flow, bacteria can grow slower because metabolism is reduced. Cryotherapy has been shown to reduce inflation, but data on this matter is controversial [13]. Despite the diversity of parameters and methodologies used for each study, comparing published pharmacological studies is difficult [39]. In maxillofacial surgery, steroids are often used. Besides steroids after wisdom tooth extraction, very few studies have been published on the potential benefits of steroids in orthognathic surgery [40, 41]. Nonsteroidal anti-inflammatory drugs (NSAIDs) may also reduce inflammation and pain. NSAIDs are often less effective than combining them with another drug in controlling post-operative pain and swelling after head and neck surgery [42]. The use of any medication may reduce post-operative side effects and have severe consequences and effects as well. As drug allergies and side effects related to drugs have increased, alternative methods have become more popular. An experiment was conducted to determine the effect of manual lymphatic drainage (MLD) following third molar extraction. Measurements of repeated face measurements demonstrated that MLD improves lymphatic circulation and reduces swelling and post-operative pain [6].

Individual influences have been eliminated from this procedure thanks to the split-mouth design, resulting in more accurate interpretations of the results. This procedure was used in a study by Heras et al. (2019), which looked at the influence of this method on the rate of edema and pain after wisdom tooth surgery, with comparable results. These differences may arise from the nature of orthognathic surgery, trauma to the jaw's sensory nerve trunks, or the injection of analgesics and steroids in the post-operative period [43]. In a study designed for a clinical trial in 2021, Jaron et al. also reported this method's effect on pain, edema, and mouth opening after bilateral wisdom tooth surgery and reported similar results. This follow-up time is appropriate because the study was only completed on days 3 and 7, and wisdom tooth surgery is a minor procedure. More complex surgeries, including jaw fractures and orthognathic surgery, require a more extended follow-up [44].

The results of this study follow those of Tozzi et al.’s 2015 study; [45]. However, the 2015 study assessed the edema rate using three-dimensional methods. The rate of edema was compared to 3D scanning methods in the 2020 study by Kocer et al., and employing more complex methods does not always lead to more accurate conclusions [46]. In addition, in a study by Lietz-Kijek et al., the study’s number and design differed from the current study’s results [47]. The purpose of this study was divided into two parts: split-mouth and a clinical trial to evaluate the effectiveness of Kinesio tape on decreasing edema in the post-operative period and increasing the rate of mouth opening after 5 days. This method can increase the speed of recovery and reduce morbidity, allowing patients to return to normal activities sooner and avoid long-term recovery’s direct and indirect expenses.

## Conclusion

Kinesio tape treated lymphatic areas, reduced tension, and reduced swelling. In the healing process, blood and lymph microcirculation were indirectly improved. This technique can be employed in various surgical ways in the facial area, in addition to orthognathic surgery, which must be explored with a more precise design and less bias in the future.

## Data Availability

Not applicable.
